# Infection Rates Following Trans-Kambin Oblique Lateral-Posterior Lumbar Interbody Fusion and Associated Procedures: A Review of 2,069 Cases

**DOI:** 10.7759/cureus.110986

**Published:** 2026-06-16

**Authors:** Hamid Abbasi, Muhammad Sarwar, Abdul Rauf, Dominic Moore

**Affiliations:** 1 Spine Surgery, Avicenna Technical University (ATU) and Inspired Spine, Minneapolis, USA; 2 Spine Surgery, Punjab Institute of Neurosciences, Lahore, PAK; 3 Neurosurgery, Inspired Spine Health, Minneapolis, USA; 4 Spine Surgery, Inspired Spine Health, Minneapolis, USA

**Keywords:** complications of spine surgery, infection rate, lumbar spine infection, minimally invasive fusion oblique lateral fusion surgical site infection trans-kambin approach retrospective cohort study, trans-kambin approach

## Abstract

Low back pain remains one of the leading causes of disability worldwide and continues to impose a substantial burden on healthcare systems. Lumbar fusion procedures have increased significantly over recent decades due to an aging population, expanded surgical indications, and advances in minimally invasive surgical (MIS) techniques. Compared with traditional open approaches, MIS lumbar fusion techniques have demonstrated reduced tissue disruption, improved recovery, and lower complication rates. Among postoperative complications, surgical site infection (SSI) remains one of the most clinically significant because of its association with increased morbidity, prolonged hospitalization, reoperation, and increased healthcare costs.

Although infection rates following traditional lumbar fusion procedures are well documented, large-scale studies specifically evaluating infection rates following minimally invasive trans-Kambin oblique lateral lumbar interbody fusion (OLLIF) remain limited. Early studies have suggested that MIS approaches may reduce the risk of postoperative infection by decreasing soft-tissue disruption and intraoperative exposure. In addition, patient-related factors such as diabetes mellitus, smoking, and elevated body mass index (BMI) are recognized contributors to postoperative infectious complications.

In this series of more than 2,069 consecutive minimally invasive lateral and oblique interbody fusion procedures performed over a 14-year period, the overall postoperative infection rate was low at 0.39%, with deep infections occurring in 0.11% of cases. When analyzed by technique, pure minimally invasive OLLIF procedures demonstrated an exceptionally low infection rate of 0.24%, whereas cases requiring an additional open posterior component had a significantly higher infection rate of 1.83% (OR = 7.66; p = 0.009). These findings suggest that infection risk was primarily associated with open exposure rather than the OLLIF technique itself. Additional factors associated with increased infection risk included revision surgery, multilevel constructs, obesity (BMI > 30 kg/m²), and elevated HbA1c levels. The two deep infections appeared to result from different mechanisms, including likely hematogenous spread and a true postoperative SSI. Overall, these findings support a favorable infection profile for the minimally invasive trans-Kambin OLLIF approach, with a markedly increased risk when open posterior components are introduced.

## Introduction

Low back pain continues to be a leading cause of disability worldwide, imposing a substantial burden on individuals and healthcare systems alike. For patients whose symptoms persist despite conservative management, spinal fusion remains a cornerstone of surgical treatment for a range of degenerative pathologies. Over time, the volume of lumbar fusion procedures has increased substantially, driven in part by an aging population and broader surgical indications. This growth has been supported by advancements in surgical techniques, which have evolved from traditional open approaches to a variety of minimally invasive surgical (MIS) options [1]. Although relatively recent, these techniques have already demonstrated improved clinical outcomes and reduced economic burden on healthcare systems [2].

Because MIS approaches aim to reduce the morbidity associated with extensive tissue dissection, their complication profiles remain a central focus of outcomes research. One of the most important complications following lumbar fusion is surgical site infection (SSI), whether involving superficial or deep tissues. These infections significantly increase patient morbidity, prolong hospitalization, increase healthcare utilization, and often necessitate additional interventions [3].

Reported infection rates following spinal fusion vary widely across studies, reflecting differences in patient populations, surgical techniques, diagnostic criteria, and follow-up duration. One study reported that, among patients undergoing single-level fusion, approximately 5.2% experienced a postoperative infection, with 2.6% classified as true SSIs and the remainder representing non-SSI infectious complications, such as urinary tract infections and pneumonia [4]. A large, separate cohort study encompassing more than 188,000 traditional lumbar fusion procedures reported an overall postoperative infection rate of approximately 10%, including superficial and deep infections, as well as systemic infections [5].

Early retrospective studies have demonstrated significantly lower SSI rates following MIS procedures compared with traditional lumbar fusion techniques. While postoperative infection rates in traditional cohorts have approached 10%, MIS procedures have been associated with substantially lower SSI rates, suggesting that reduced soft-tissue disruption contributes to a decreased risk of infection (approximately 0.5% for MIS versus 3.3% for open techniques overall) [6-10].

Multiple perioperative patient factors are recognized as important determinants of postoperative outcomes, as emphasized by the Congress of Neurological Surgeons (CNS) evidence-based guidelines for perioperative spine care. These factors include: (1) diabetes mellitus, as patients with a preoperative hemoglobin A1c (HbA1c) level greater than 7.5% are at increased risk of SSI and reoperation; (2) smoking, as active smokers have an elevated risk of reoperation, although evidence supporting preoperative smoking cessation is limited and counseling to abstain remains recommended; and (3) elevated body mass index (BMI). Although evidence regarding BMI and the risk of SSI or reoperation remains conflicting, patients with a BMI > 30 kg/m² should be counseled regarding the potential increased risk. However, no specific BMI threshold is currently recommended [11].

Despite extensive literature on infection rates following lumbar fusion, comparatively little large-scale evidence specifically addresses minimally invasive lateral fusion variants such as trans-Kambin oblique lateral lumbar interbody fusion (OLLIF). Understanding how SSI rates following MIS-OLLIF compare with those reported in broader lumbar fusion cohorts is essential for contextualizing the safety and complication profile of this increasingly utilized technique. OLLIF can be safely performed across multiple levels, from T12 to S1, and may even be performed in an outpatient setting [7].

A planned limited open posterior exposure was performed in selected patients with prior instrumentation to facilitate safe hardware connection, revision, or removal while maintaining a minimally invasive surgical strategy. This technique was used only when existing implants could not be adequately accessed or addressed through minimally invasive methods alone and was analyzed as a separate intraoperative variable.

## Materials and methods

Study design

This retrospective cohort study included 2,069 lumbar fusion procedures performed between March 2012 and December 2025. Of these, 1,861 were performed using the MIS-OLLIF technique [1]. The remaining cases consisted of technically similar minimally invasive lateral fusion approaches, including direct lateral interbody fusion (MIS-DLIF; n = 181) and oblique thoracic interbody fusion (MIS-OTIF; n = 27). Procedures that included an open component (n = 218) were performed using a modified Wiltse approach, which is designed to preserve paraspinal musculature through linear splitting of the multifidus and longissimus muscles [2].

All procedures were performed by a single surgeon, ensuring technical consistency across the study cohort. Surgeries were conducted at the following institutions: Douglas County Hospital (now Alomere Health), Alexandria, MN; Riverview Health, Crookston, MN; Fairview Ridges Hospital, Burnsville, MN; Maple Grove Surgery Center, Maple Grove, MN; Inspired Spine Surgery Center, Burnsville, MN; and Summit Surgical, Hutchinson, KS.

Patient selection

All patients underwent a comprehensive preoperative evaluation prior to surgical consideration [3]. Imaging studies included lumbar spine MRI and standing lumbar radiographs with flexion-extension views. Additional diagnostic studies, such as computed tomography (CT) and provocative discography, were obtained when clinically indicated.

Indications for OLLIF included severe degenerative disc disease [4], spondylolisthesis [5], discogenic pain, foraminal stenosis, recurrent disc herniation, and, in selected cases, correction of scoliosis or other spinal deformities. All patients had completed an adequate course of conservative management prior to surgical consideration, including but not limited to physical therapy, pharmacologic treatment, and/or injection therapy.

Outcome measures

The primary outcome was postoperative infection following spine surgery, classified according to the timing of presentation. Early infection was defined as infection occurring within three months postoperatively and typically represented SSIs related to perioperative contamination, wound-healing complications, or early hardware-associated infection. These infections commonly present with wound erythema, drainage, pain, fever, or elevated inflammatory markers.

Delayed infection was defined as infection occurring between 3 and 12 months postoperatively. This category often reflects indolent or low-grade infections, frequently associated with biofilm formation on spinal instrumentation. Delayed infections may present with persistent back pain, subtle wound abnormalities, implant loosening, or progressive inflammatory changes in the absence of overt systemic signs.

Late infection was defined as infection occurring more than 12 months after surgery. These infections are uncommon and are typically associated with hematogenous seeding, late hardware-associated infection, or underlying chronic osteomyelitis or discitis. Clinical presentation may include recurrent pain, systemic symptoms, or radiographic evidence of bony or disc space infection.

Patients were followed through routine postoperative clinical encounters. All documented visits, communications, and follow-up records within the first postoperative year were reviewed for evidence of infectious complications. Although surveillance extended through one year postoperatively, no cases of delayed or late deep infection, including osteomyelitis or discitis, were identified.

Data collection

Clinical data were retrospectively obtained from the relevant electronic medical record (EMR) systems of participating institutions. Data collected included body mass index (BMI) [10], operative time [11], procedure type, complications, hemoglobin A1c (HbA1c), where available [12], and smoking status [13]. Extracted data were stored in a secure, access-restricted database, with regular activity logging to ensure data integrity and confidentiality.

Procedures were classified as either pure minimally invasive surgery (MIS) [14,15] or hybrid procedures involving MIS combined with open posterior instrumentation, reinstrumentation, or hardware removal. Revision surgery [16] was defined as reinstrumentation or hardware removal at previously instrumented levels.

All postoperative infections were reviewed by the operating surgeon and classified according to the Centers for Disease Control and Prevention (CDC) surgical site infection (SSI) criteria. Infections were categorized as follows: (1) superficial incisional SSI, defined as involvement limited to the skin or subcutaneous tissue above the fascial plane; (2) deep incisional SSI, defined as involvement of the fascial and muscle layers; (3) deep tissue infection, defined as involvement of any anatomic structure deeper than the fascia, including epidural abscesses or vertebral osteomyelitis; and (4) non-SSI infection, defined as cases with equivocal documentation or infections likely attributable to non-surgical etiologies (e.g., hematogenous seeding), which were excluded from the formal SSI analysis.

Statistical analysis

Infection rates are reported as percentages with 95% Wilson score confidence intervals. Categorical comparisons (open versus MIS approach, revision versus primary surgery, multilevel versus one- or two-level procedures, and BMI category) were performed using Fisher’s exact test, with odds ratios (ORs) and exact p-values reported. Continuous variables (BMI and operative time) in the infected cohort were compared with population means using one-sample t-tests. All statistical tests were two-tailed, with statistical significance defined as p < 0.05.

## Results

The study cohort consisted of 2,069 procedures: 1,861 OLLIF (89.9%), 181 DLIF (8.7%), and 27 OTIF (1.3%). Of the OLLIF procedures, 1,643 (88.3%) were completed using a purely MIS approach, whereas 218 (11.7%) included an open posterior component, primarily to access previously implanted hardware. Mean BMI was 31.8 kg/m² (range, 17-64; data available for 1,817 of 1,861 cases), and mean operative time was 69.6 minutes (range, 12-289; data available for 1,829 of 1,861 cases).

A total of eight postoperative infections (0.43%; 95% confidence interval (CI), 0.22-0.85%) were reported among the 1,861 procedures performed using the OLLIF approach. No infections were reported among the 181 DLIF or 27 OTIF procedures. Across the entire cohort, the overall infection rate was 0.39% (8/2,069; 95% CI, 0.20-0.76%). Of the eight reported infections, four met formal CDC criteria for SSI, whereas four demonstrated documented infection-related clinical features but did not meet strict SSI definitions. Six infections (75.0%) were classified as superficial skin infections (0.32%; 95% CI, 0.15-0.70%), and two (25.0%) were classified as deep wound infections (DWIs) (0.11%; 95% CI, 0.03-0.39%), as shown in Table 1.

**Table 1:** Primary infection groups. This table shows infection rates for superficial skin infections (SfSI) and deep wound infections (DWI) in relation to all cases and oblique lateral lumbar interbody fusion (OLLIF) cases (both pure minimally invasive surgical (MIS) and hybrid open procedures).

Group	Infections/total	Rate (%)	Confidence interval (95%)
All procedures	8/2069	0.39	0.20%-0.76%
SSIs	6/2069	0.29	0.13%-0.63%
DWIs	2/2069	0.1	0.03%-0.35%
All OLLIFs	8/1861	0.43	0.22%-0.85%
All OLLIF SfSIs	6/1861	0.32	0.15%-0.70%
All OLLIF DWIs	2/1861	0.11	0.03%-0.39%

Table 2 summarizes subgroup analyses of infection rates stratified by surgical approach, revision status, and number of fused levels. Results are presented as both absolute counts and percentages. Purely minimally invasive OLLIF procedures demonstrated the lowest infection rate at 0.24% (4/1,643) and served as the reference group, whereas procedures involving an open posterior component had an infection rate of 1.83% (4/218). Revision or reinstrumentation cases demonstrated an infection rate of 1.66% (3/181), compared with 0.30% (5/1,680) among primary procedures. Similarly, multilevel constructs (>2 levels) demonstrated a higher infection rate of 1.13% (4/353), compared with 0.27% (4/1,508) in single- or two-level procedures. ORs were calculated using Fisher’s exact test, with corresponding p-values reported in Table 2.

**Table 2:** Infection rates stratified by surgical approach, revision status, and number of levels. MIS-OLLIF: minimally invasive oblique lateral lumbar interbody fusion.

Subgroup	Infections/total	Rate (%)	OR	p-value
Purely MIS-OLLIF	4/1,643	0.24	Ref.	-
OLLIF + open component	4/218	1.83	7.66	0.009
Primary procedures	5/1,680	0.3	Ref.	-
Revision/reinstrumentation	3/181	1.66	5.65	0.035
Single/two-level	4/1,508	0.27	Ref.	-
Multi-level (>2 levels)	4/353	1.13	4.31	0.047

Table 3 presents infection rates stratified by BMI category. The mean BMI of infected patients was 38.8 kg/m² (SD, 8.6; range, 29-53), compared with 31.8 kg/m² in the non-infected cohort (p = 0.059). Mean operative time for infected cases was 78.0 minutes (range, 32-128), whereas the mean operative time for the overall cohort was 69.6 minutes (p = 0.506). A dose-response relationship was observed between increasing BMI category and infection rate. No infections occurred among patients with normal weight (0.00%). Infection rates increased progressively to 0.19% among overweight patients, 0.39% among patients with class I obesity, and 0.65% among patients with class II obesity. The highest infection rate was observed among patients with class III obesity, at 1.25%.

**Table 3:** Infection rates stratified by the WHO body mass index category. This table shows infection rates for superficial skin infections (SfSI) and deep wound infections (DWI) in relation to all cases and oblique lateral lumbar interbody fusion (OLLIF) cases (both pure minimally invasive surgical (MIS) and hybrid open procedures).

BMI category	Total n	Infections	Rate (%)	p-value vs. normal/overweight
Normal (<25)	235	0	0	-
Overweight (25-29.9)	517	1	0.19	Ref
Obese class I (30-34.9)	515	2	0.39	0.61
Obese class II (35-39.9)	310	2	0.65	0.26
Obese class III (≥40)	240	3	1.25	0.077

Infection case characteristics are summarized in Table 4. Infections did not occur in a clustered pattern over time, with one case each reported in 2017, 2020, 2021, 2022, and 2023; two cases reported in 2024; and one case reported in early 2025. Annual infection rates ranged from 0.0% to 0.9%, with no statistically significant temporal trend identified.

**Table 4:** Characterization of all eight infection cases. This table shows the status information on each of the eight reported infections, including date of the infection, procedure performed, patient BMI, presence of elevated HbA1C, smoking status, operation time, type of infection, and a brief description of management. OLLIF: oblique lateral lumbar interbody fusion, MIS: minimally invasive surgical technique, I&D: incision and drainage, HW: hardware, SSI: superficial skin infections, DWI: deep wound infections.

Case	Date	Procedure	BMI	HbA1C > 7.5 mg/dL	Smoking	OpTime	Classification	Management
1	09/2017	L3-S1 OLLIF	29	-	-	96	SfSI	Oral antibiotics
2	08/2020	L5-S1 OLLIF + open L4-S1	31	-	-	91	SfSI	I&D bedside, local anesthesia
3	10/2021	L5-S1 OLLIF	33	-	-	32	SfSI	Oral antibiotics
4	05/2022	L3-S1 OLLIF	35	-	-	66	SfSI	Oral antibiotics
5	10/2023	L4-L5 OLLIF + open L5-S1 reinstrumentation	50	-	-	128	SfSI	Oral antibiotics
6	11/2024	T12-L2 OLLIF + open L2-L3 + HW removal	40	+	+	81	SfSI	Wound care, oral antibiotics
7	12/2024	L4-S1 OLLIF (pure MIS)	53	+	-	52	DWI	I&D in OR, IV antibiotics
8	01/2025	L2-L4 OLLIF + L5-S1 revision + open reinstrumentation	39	+	-	-	DWI	I&D in OR, IV antibiotics

## Discussion

Of the eight infections identified in this series, two were deep infections and warrant closer examination because they represent significant postoperative complications. The first patient (Case 7) underwent a relatively straightforward L4-S1 OLLIF procedure lasting 52 minutes. Postoperatively, the patient presented to the emergency department with leukocytosis (WBC 17.4 × 10⁹/L). By postoperative day 10, the WBC count had normalized; however, imaging revealed an epidural abscess at L3-4, one level above the surgical site and within a previously uninstrumented segment. There was no involvement of the surgical wound or instrumentation, suggesting hematogenous seeding from an alternative source [10,13]. Notably, the patient had a history of postoperative infections following prior surgical procedures, including knee surgery, without an identified infectious source despite a comprehensive medical evaluation, including rheumatologic workup. The patient also had significant risk factors, including severe obesity (BMI 53 kg/m²), underscoring the importance of perioperative metabolic optimization [3,4,6]. The infectious process was likely influenced by a combination of patient-specific factors, prior susceptibility to infection, and underlying comorbidities [3,10].

The second deep infection (Case 8) occurred in a patient who underwent L2-L4 OLLIF with L5-S1 revision and open posterior reinstrumentation. This patient was at elevated risk because of obesity (BMI 39 kg/m²) and poorly controlled diabetes mellitus, with an HbA1c > 7.5% [3,4,6]. The infection presented as a deep posterior abscess at the instrumentation site without systemic signs such as fever or leukocytosis. In contrast to Case 7, this infection was likely related to the open posterior exposure and revision instrumentation rather than the OLLIF technique itself [9,12,16]. Imaging played a critical role in diagnosis because clinical findings were subtle [6,13].

Among the deepest infections in this cohort, several factors were associated with increased infection risk: open posterior approach (OR = 7.66; p = 0.009), revision/reinstrumentation (OR = 5.65; p = 0.035), and multilevel construct (OR = 4.31; p = 0.047).

Class II-III obesity and elevated HbA1c levels were present in the deep infection cases (Cases 7 and 8), further suggesting a potential contribution of patient-specific metabolic factors to postoperative infection risk [3,4,6]. However, as shown in Table 3, BMI was not significantly associated with infection risk in OLLIF and related procedures (p > 0.05), although infection rates appeared to increase progressively among patients with BMI values exceeding 40 kg/m². Elevated HbA1c levels were also observed in these deep infection cases, consistent with CNS guideline-based risk stratification [3].

Case 7, involving an epidural abscess at a non-instrumented level, is most consistent with hematogenous seeding, a mechanism previously described in the literature [10,13]. In contrast, Case 8, involving a deep posterior abscess at the instrumentation site following revision surgery, represents a true SSI and highlights the importance of postoperative surveillance in higher-risk patients [9,12,16].

These findings suggest that patients can be counseled that the risk of infection following a purely minimally invasive OLLIF procedure is very low (0.24%) [1,15]. However, when an open posterior component is required, even through a muscle-sparing Wiltse approach, infection risk increases to 1.83% (OR = 7.66; p = 0.009) [3,9,14]. Although both approaches are designed to minimize tissue disruption, the additional surgical exposure associated with open procedures likely contributes to the observed increase in infection risk. Patients with obesity, diabetes mellitus, or elevated HbA1c levels may benefit from preoperative optimization strategies, including improved glycemic control and weight reduction when feasible [3,4,6]. Given the observed association between metabolic risk factors and infection, future research may investigate whether emerging pharmacologic therapies that facilitate weight loss can improve perioperative outcomes and influence the management of lumbar spinal pathology.

Several limitations should be considered when interpreting these findings. Information regarding diabetes management and smoking status among the 2,061 patients without infection was incomplete, limiting further analysis of these potential risk factors. In addition, all procedures were performed by a single surgeon. Although this enhances technical consistency, it limits the generalizability of the findings to other surgeons and practice settings.

Overall, these limitations highlight areas for future investigation, particularly regarding external validity and the interpretation of risk factor associations in a cohort with a limited number of infection events. Future multicenter studies and analyses incorporating outcomes from additional surgeons, including others experienced with the OLLIF technique, may further strengthen the generalizability and reproducibility of these findings.

## Conclusions

In this large consecutive series of minimally invasive OLLIF procedures performed over a 14-year period, the OLLIF technique demonstrated a favorable infection profile, with a low incidence of postoperative infectious complications. Purely minimally invasive OLLIF procedures were associated with a lower risk of infection than cases requiring an additional open posterior component, suggesting that increased soft-tissue exposure may contribute substantially to postoperative infection risk.

Higher infection rates were observed in association with revision surgery, multilevel constructs, obesity, and poor glycemic control. The occurrence of both hematogenous infections and postoperative SSIs further underscores the importance of careful postoperative surveillance and individualized patient assessment. Overall, these findings support the safety of the minimally invasive trans-Kambin OLLIF approach and highlight the potential benefits of minimizing open surgical exposure and optimizing modifiable patient risk factors to reduce postoperative infectious complications.
